# Bacterial migration through punctured surgical gloves under real surgical conditions

**DOI:** 10.1186/1471-2334-10-192

**Published:** 2010-07-01

**Authors:** Nils-Olaf Hübner, Anna-Maria Goerdt, Natalie Stanislawski, Ojan Assadian, Claus-Dieter Heidecke, Axel Kramer, Lars Ivo Partecke

**Affiliations:** 1 Institute of Hygiene and Environmental Medicine, Ernst-Moritz-Arndt University, Greifswald, Germany; 2 Department of Surgery, Clinic of General, Visceral, Vascular and Thoracic Surgery, Ernst-Moritz-Arndt University, Greifswald, Germany; 3 Department of Hygiene and Medical Microbiology, Medical University of Vienna, Vienna, Austria

## Abstract

**Background:**

The aim of this study was to confirm recent results from a previous study focussing on the development of a method to measure the bacterial translocation through puncture holes in surgical gloves under real surgical conditions.

**Methods:**

An established method was applied to detect bacterial migration from the operating site through the punctured glove. Biogel™ double-gloving surgical gloves were used during visceral surgeries over a 6-month period. A modified Gaschen-bag method was used to retrieve organisms from the inner glove, and thus-obtained bacteria were compared with micro-organisms detected by an intra-operative swab.

**Results:**

In 20 consecutive procedures, 194 gloves (98 outer gloves, 96 inner gloves) were examined. The rate of micro-perforations of the outer surgical glove was 10% with a median wearing time of 100 minutes (range: 20-175 minutes). Perforations occurred in 81% on the non-dominant hand, with the index finger most frequently (25%) punctured. In six cases, bacterial migration could be demonstrated microbiologically. In 5% (5/98) of outer gloves and in 1% (1/96) of the inner gloves, bacterial migration through micro-perforations was observed. For gloves with detected micro-perforations (n = 10 outer layers), the calculated migration was 50% (n = 5). The minimum wearing time was 62 minutes, with a calculated median wearing time of 71 minutes.

**Conclusions:**

This study confirms previous results that bacterial migration through unnoticed micro-perforations in surgical gloves does occur under real practical surgical conditions. Undetected perforation of surgical gloves occurs frequently. Bacterial migration from the patient through micro-perforations on the hand of surgeons was confirmed, limiting the protective barrier function of gloves if worn over longer periods.

## Background

During surgery, intact surgical gloves act as a physical barrier against the transmission of blood-borne pathogens from hospital staff to patients and vice versa [1]. Several studies demonstrated that unrecognized perforations of surgical gloves are not uncommon and that the frequency of defective gloves increases with duration of wear [2,3]. It was also shown that the risk of perforations depends on the type of surgery performed, ranging from 7% in urological surgery up to 65% in cardiac surgery [4-8].

In a previous study, we established a method to evaluate the quantity of bacteria passing through undiscovered glove punctures under real surgical conditions [9]. We demonstrated that during surgery, micro-perforations allow the passage of large quantities of bacteria from the surgical site through the punctures.

The purpose of this prospective investigation was to confirm our previously published results on bacterial translocations [9].

## Methods

During a period of six consecutive months from December 2007 to May 2008, gloves from a total number of 20 elective and emergency surgical laparotomies in the Department of General Surgery at the Ernst-Moritz-Arndt University, Greifswald, were analyzed. The indications for laparotomy included: perforations and resections of the gastrointestinal tract (n = 17) and lavages for underlying peritonitis (n = 3). Detailed demographic data of the procedures included are summarized in table 1. All members of the scrub team used powder-free double-gloving gloves with a puncture indication system (Biogel^® ^Indicator™, Mölnycke, Gothenburg, Sweden). This patented indicator system gives visual warning of glove puncture in the presence of fluid by showing a dark green patch around the site of puncture and has been shown to effectively indicate punctures [10].

**Table 1:** Type and duration of operations included and number of gloves assessed per operation

type of operation	duration	type	gloves worn
rectum/sigma resection	108 min	colorectal	8

relaparatomy, purulent situs	57 min	emergency	4

Rectum resection	184 min	colorectal	9 (5/4)*

Rectum resection	155 min	colorectal	8

hemicolectomy	109 min	colorectal	8

hemicolectomy	74 min	colorectal	13 (7/6)*

extended hemicolectomy	150 min	colorectal	16

hemicolectomy right	100 min	colorectal	16

Sigma resection	177 min	colorectal	8

partial resection of colon	232 min	colorectal	4

Rectum amputation	171 min	colorectal	4

hemicolectomy/rectum resection	99 min	colorectal	12

replacemant of ileostomy	61 min	colorectal	12

Rectum amputation	144 min	colorectal	12

Rectum amputation	150 min	colorectal	12

hemicolectomy	62 min	colorectal	12

Sigma resection	297 min	colorectal	16

relaparatomy	85 min	emergency	8

revision colon fistula	161 min	emergency	4

hemicolectomy	109 min	colorectal	8

	**Median 126.5 min**		**∑ = 194**

* outer/inner gloves; in these cases, only the outer glove was changed due to the intraoperative situation

There was no directive to surgeons on maximum glove wearing time. Gloves were changed and examined when a perforation was detected by the indicator system or at the end of a procedure. The impermeability of all inner and outer gloves worn was tested according to DIN EN 455-1 immediately after sampling. Gloves with obvious macro-perforations at any time were excluded. The person wearing the glove, their role within the surgical team, the type of surgery, date and the wearing time were documented. Surgical sites were examined by taking one swab from an area inside the situs and at the moment assumed to be most heavily contaminated, and was further processed following standard microbiological methods.

To investigate bacterial migration from patients to the hand of the surgical staff through micro-perforations in gloves, the modified and standardized Gaschen-Bag method was used as previously described [9]. Briefly, after removal of the outer glove, the hand with the inner glove was shaken out in a sterile plastic bag ("Gaschen-Bag") containing 100 ml sterile NaCl 0.9%, which was then processed by membrane filtration. Maximum care was taken to prevent transmission of organisms to the inner glove when removing the outer glove. A scrub nurse and an investigator assisted in glove removal according to a standard procedure. Transmission was considered as proven if identical bacteria (species and antibiogram) were obtained from the swab and the inner glove. The examined microorganisms excluded anaerobic bacteria, spores and viruses.

## Results

**Figure 1:**
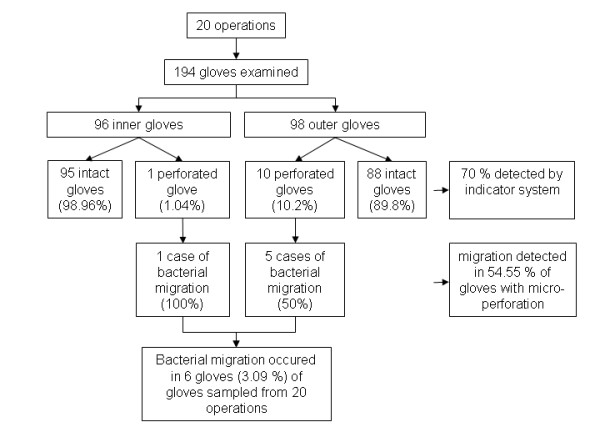
**Gloves, perforations/migration detected and performance of the indicator system**.

In 20 consecutive surgical operations, 194 gloves (98 outer gloves, 96 inner gloves) were examined [Figure 1]. All participants were right handed. In two cases, only the outer gloves were changed because the situation did not allow a longer interruption of the procedure. The median wearing time was 99.6 minutes (range 20 to 175 minutes). A total of 10 (10.2%) of the outer gloves and 1 (1.04%) of the inner gloves were found to be perforated. Thirteen (81.3%) of perforations occurred in the left outer layer, while the index finger of the non-dominant hand was found to be the most commonly affected location of perforations in 4 gloves (25%). The 10 perforated outer gloves showed 15 (7 × 1 perforation, 1 × 2 perforations, 2 × 3 perforations) and the one perforated inner layer showed only one perforation. In 7 (70%) of all worn gloves, the perforation was noticed by the surgical team members because the indicator system showed a color change. In six cases, bacterial migrations were demonstrated microbiologically. In 5 (5.1%) of the examined outer gloves (n = 98) and in 1 (1.04%) of the examined inner gloves (n = 96), bacterial migration through micro-perforations was shown: the same bacteria were detected in the swab and the Gaschen-Bag (or were typical organisms of the residual skin flora in the case of the perforated inner glove) (Illustration 1). Pertaining to gloves with detected micro-perforations (n = 10 outer layers), the calculated migration was 50% (n = 5). The minimum wearing time was 62 minutes with a calculated median wear duration of 71 minutes [Table 2]. Among the migrated bacteria, *Micrococcus luteus*, *enterococci *and *E. coli *were identified.

**Table 2:** Migration of bacteria from the operation site through punctures in surgical gloves

Number	Surgical team member	Duration of wear (minutes)	Location/site of perforation	organisms found in the glove	organisms found in the wound
1	surgeon	72	Index finger, right*	CNS	CNS, Strep. spp.

2	surgeon	65	between ring and little finger, left*	E. coli, Proteus penneri	E. coli, Proteus penneri, CNS

3	surgeon	90	Index finger, left*	CNS	CNS

4	surgeon	62	Thumb, left*	E. coli strain A and B	E. coli strain A, B and C

5	surgeon	70	Index finger, left*	Enterococcus cloacae	Enterococcus cloacae

6	surgeon	72	Thumb, left**	M. luteus	CNS, Strep. spp.

Number 1-5: bacterial migration through puncture of outer glove; 6: bacterial migration from surgeon's hand through puncture of inner glove; * outer glove punctured, ** inner glove punctured

## Discussion

The intact surgical glove is the most important barrier to protect the patient from microorganisms from the hand of the surgical team and vice versa. The preoperative surgical hand preparation can reduce but not eradicate the resident flora on the surgeon's hands, therefore reducing but not eliminating the risk of transmission of resident organisms into the wound. Conversely, blood-borne pathogens can be transmitted from the patient to the surgeon and endanger surgical team members [11].

The role of glove perforation as risk factor of surgical site infection (SSI) is still not well understood. In a recently published study conducted by Misteli et al., macro-perforations of surgical gloves were found to be a significant risk factor for the development of SSI in cases where prophylactic antibiotics were not administered [11].

Neither the question if of micro-perforations compromise the aseptic barrier nor as a risk factor for SSI has been well studied yet. To our knowledge, Harnoss et al. were the first who described the translocation of microorganisms through undetected micro-perforations under real conditions [9]. Our study reconfirms these results: For gloves with perforations, the calculated migration rate was as high as 54.5% (6 cases out of 11 gloves total). Again, we were able to show that micro-perforations breach the aseptic barrier. This implies that micro-perforations could equal macro-perforations as a risk factor for SSI. This is supported by the findings of Al Maiyah et al., who demonstrated that a routine change of gloves during orthopedic surgery can significantly reduce the risk of bacterial migration through reduction of unnoticed glove perforation [12].

Interestingly, only the surgeon was affected in this study, with his index fingers and thumbs/middle fingers at major risk for bacterial migration [Table 2]. Results from recently published studies demonstrate that the majority of perforations occurred on non-dominant hands, which agrees with our findings [2,9,13-18].

As we showed previously, the incidence of micro-perforations in surgical gloves depends on the duration of wear [2]. We demonstrated that the risk is high of micro-organisms passing through undetected micro-perforations in surgical gloves during surgery. Since the rate of micro-perforations increases with the duration of wear, gloves should be changed at least every 90 minutes to maintain a safe barrier layer. Alternatively, the barrier function of gloves can be improved by double-gloving or by strengthening the gloves at sites pre-disposed to perforation.

The results from that study clearly indicate that the risk of micro-perforations and, as a consequence, the loss of protection increases with wear duration. Based on these data, a glove change for the surgeon and the first assistant after 90 minutes and after 150 minutes for the second assistant and the scrub nurse was recommended by the authors. In the meantime, these recommendations have been adopted be the Association of the Scientific Medical Societies in Germany (AWMF) [19].

While our study has several limitations, it encourages safety measures to lower the risk of glove perforation. The possible transmission of pathogens shown here adds only indirect evidence to the role of micro-perforations as a risk factor for SSI. Because this was a single-center study using a single glove brand and including only one type of surgery, the results may not be fully transferable to other settings or glove brands. Essentially, the frequency of micro-perforations, the percentage of translocations, and the relation between wear duration and the number of perforations may vary. Further research should include multicenter studies with clinical endpoints to confirm our results. Nonetheless, high frequencies of perforations in surgical gloves have been repeatedly described in literature, and our findings are supported by results from other groups and settings, as well as by our own group [1,3-6,8,12-17].

Due to ethical and safety considerations, the recommendations given by Partecke et al. and the AWMF for the daily routine in surgical settings should be followed even before final proof is available. These recommendations include a change of gloves after 90 minutes. While our findings support an earlier change, this seems to be a good compromise between safety and feasibility [2,18].

To improve protection of the whole surgical team and the patient, an alternative for changing gloves after 90 minutes might be an improvement of glove material or the application of double gloving [19]. This becomes particularly important when maximum sterility is required (as with joint replacements), the patient is known to have a blood-borne disease, or there is a high risk of damaging the glove (as in bone surgery). An additional, second pair of gloves (double gloving) significantly reduces the incidence of micro-perforations to the inner glove [18]. Moreover, an indicator glove could be used. A change of color indicates a perforation and the glove can then be changed even prior to 90 minutes if necessary [4,10,16]. In the present study, 70% of the perforations (of the outer gloves) could be detected by the indicator-glove system. This, together with the fact that only one of all tested inner gloves was punctured in this study, indicates that the double-gloving indicator system provides reliable prevention of transmission of microorganisms.

## Conclusions

This study confirms previous results that bacterial migration through unnoticed micro-perforations in surgical gloves does occur under real practical surgical conditions. Undetected perforation of surgical gloves occurs frequently. Bacterial migration from the patient through micro-perforations onto the hand of surgeons was confirmed, limiting the protective barrier function of gloves if worn over longer periods. Preventive measures for lowering the risk of glove perforation can include a change of gloves at least every 90 minutes, the use of double-gloving, or the specific strengthening of predilection sites for punctures, and are therefore strongly recommended.

## Competing interests

The authors declare a financial competing interest:

Mölnycke Healthcare, Gothenburg, Sweden accepted the costs of the study including the article-processing charge.

## Authors' contributions

NOH had the idea for the study and planned and supervised the experiments, drafted the manuscript, and analyzed and interpreted the data. AMG participated in the design of the study and helped to draft the manuscript. NS performed most of the microbiological methods and helped to draft the manuscript. OA participated in the design of the study and helped to draft the manuscript. CDH participated in the study design and coordination, and helped to draft the manuscript. AK participated in the study design and coordination, and helped to draft the manuscript. LIP participated in the design of the study, supervised the experiments, analyzed and interpreted the data, and helped to draft the manuscript.

All authors have been involved in drafting the manuscript or revising it critically for important intellectual content and have read and approved the final manuscript.

## Pre-publication history

The pre-publication history for this paper can be accessed here:

http://www.biomedcentral.com/1471-2334/10/192/prepub
